# Real-world efficacy of stapokibart for severe olfactory dysfunction: early and sustained improvement independent of baseline type 2 inflammation status and nasal polyp burden

**DOI:** 10.3389/fimmu.2026.1858283

**Published:** 2026-06-11

**Authors:** Xiang-Ning Cheng, Liu-Qing Zhou, Yue Zhou, Sha-Zhou Li, Shan Chen, Qing Cheng, Li-Yue Li, Jian-Jun Chen, Tao Zhou

**Affiliations:** Department of Otorhinolaryngology, Union Hospital, Tongji Medical College, Huazhong University of Science and Technology, Wuhan, China

**Keywords:** chronic rhinosinusitis with nasal polyps, IL-4, olfactory dysfunction, stapokibart, type 2 inflammation

## Abstract

**Background:**

Olfactory dysfunction (OD) is a key feature of chronic rhinosinusitis with nasal polyps (CRSwNP), resulting from both mechanical obstruction and sensorineural injury. Stapokibart is a humanized anti-IL-4 receptor alpha antibody, and this real-world study aimed to evaluate its efficacy in CRSwNP patients with moderate-to-severe OD, as well as the impact of baseline characteristics on olfactory recovery.

**Methods:**

A total of 40 CRSwNP patients with moderate-to-severe OD were enrolled. All patients received subcutaneous stapokibart at a dose of 300 mg every 2 weeks for 16 weeks. Subjective olfactory outcome (smell loss VAS) and objective olfactory outcome (SSIT-16) were assessed at Weeks 0, 2, 4, 8, and 16. Subgroup analyses were stratified by baseline inflammatory markers and prior surgery history.

**Results:**

Stapokibart led to rapid and sustained olfactory improvement: smell loss VAS was significantly reduced by Week 2 (P<0.0001). At Week 16, 52.5% of patients achieved normosmia, with the mean objective olfactory score increasing from 3.05 ± 2.98 to 11.35 ± 3.73 (P<0.0001) and nasal polyp score decreasing from 4.03 ± 1.70 to 1.63 ± 1.61 (P<0.001). Olfactory recovery was consistent across subgroups, regardless of baseline IgE level, eosinophil count, or previous sinus surgery. Patients with higher baseline polyp burden showed faster early improvement but similar final outcomes.

**Conclusion:**

Stapokibart effectively and rapidly improves olfactory function in CRSwNP. Baseline nasal polyp burden affects the speed but not the magnitude of recovery, supporting the clinical utility of stapokibart in patients with moderate-to-severe OD.

## 1 Introduction

Chronic rhinosinusitis (CRS) represents a heterogeneous inflammatory condition of the nasal and paranasal mucosa, affecting approximately 4%–5% of the general population and imposing a substantial socio-economic burden on both individual patients and healthcare systems (1). Within the clinical spectrum of CRS, chronic rhinosinusitis with nasal polyps (CRSwNP) is a distinct phenotype predominantly driven by type 2 inflammation, accounting for approximately 25%–30% of all CRS cases (1–3). Among the various clinical manifestations of CRSwNP, olfactory dysfunction (OD) is not only a core diagnostic criterion but also one of the most debilitating symptoms impacting health-related quality of life (4). Recent epidemiological data indicate that while OD affects 46%–80% of the overall CRS population (5–7), its prevalence in CRSwNP patients is significantly higher, ranging from 56.0% to 97%, compared to only 13.7% in those without polyps (CRSsNP) (5, 6, 8–11). This profound sensory impairment severely restricts food appreciation, compromises the perception of environmental hazards, and is frequently associated with psychological distress, including anxiety and depression.

The pathogenesis of CRSwNP-related OD is recognized as a dual-pathway process. It involves both mechanical obstruction of the olfactory cleft by polyps or mucosal edema, which prevents odorants from reaching the olfactory epithelium, and sensorineural damage induced by the chronic inflammatory milieu (12, 13). Emerging evidence from mechanistic studies has identified the IL-4–IL-4R axis as a central regulator of this neuro-olfactory impairment. Experimental models demonstrate that IL-4, rather than IL-13, directly modulates the activity of olfactory sensory neurons (OSNs) and triggers neuroinflammation without necessarily compromising the structural integrity of the nasal mucosa (14). This suggests that targeted inhibition of IL-4R signaling is essential not only for reducing polyp volume but also for the direct restoration of neuroimmune homeostasis and olfactory function.

Despite the availability of traditional interventions, such as functional endoscopic sinus surgery (FESS) and corticosteroids, their efficacy in restoring olfaction remains highly variable and unpredictable (15–18). Reported olfactory improvement rates after FESS fluctuate between 0% and 97%, and a significant proportion of patients experience symptom recurrence within three to five years (19–21). In addition, surgery may exacerbate olfactory problems in some cases; therefore, it is generally not recommended when OD is the main symptom (19–21). Furthermore, the long-term use of systemic corticosteroids is limited by substantial adverse effects, such as osteoporosis and diabetes (1). The advent of biologics, particularly those targeting IL-4R like dupilumab, has marked a paradigm shift, demonstrating rapid and sustained olfactory recovery that often precedes the regression of structural polyps (14, 22, 23).

Stapokibart is a humanized anti-IL-4R monoclonal antibody that has recently been approved in China for the management of CRSwNP that is inadequately controlled by conventional therapies (24, 25). Preclinical epitope mapping has demonstrated that stapokibart binds to a distinct site on the IL-4R subunit, situated significantly closer to the ligand-binding region than the epitope targeted by dupilumab (24). This biochemical differentiation translates into potent biological activity in blocking IL-4 and IL-13 signaling, which has shown promising efficacy in improving polyp scores and nasal congestion in clinical trials (24).

However, despite these therapeutic advancements, real-world evidence regarding the efficacy of stapokibart in the early treatment phase of patients with severe OD remains sparse. Furthermore, it remains unclear how baseline inflammatory profiles, polyp burden, or clinical comorbidities consistently influence the longitudinal trajectory of olfactory recovery in a real-world setting. Therefore, this study aims to evaluate the real-world clinical efficacy of stapokibart in CRSwNP patients with severe OD, while identifying key baseline predictors of therapeutic response to facilitate personalized management strategies.

## 2 Methods

### 2.1 Study population

This real-world study was approved by the local ethics committee(approval number: UHCT250524), and all patients provided written informed consent prior to treatment initiation. A total of 40 adult patients with endoscopically confirmed CRSwNP and moderate-to-severe olfactory dysfunction were enrolled. Patient inclusion strictly followed the five criteria of the EPOS/EUFOREA 2023 guidelines (25). Two items were defined as mandatory criteria: persistent moderate-to-severe olfactory dysfunction (VAS smell loss score ≥ 5 cm) and laboratory evidence of type 2 inflammation. Type 2 inflammation was assessed by two routine peripheral biomarkers: peripheral blood eosinophils ≥ 150 cells/μL or serum total IgE ≥ 100 IU/mL. In addition, all patients met at least one of three supportive criteria: poor quality of life (SNOT-22 score ≥ 40), systemic corticosteroid dependence or contraindication, or comorbid asthma (detailed baseline characteristics in **Table 1**). Patients were excluded if they had a history of head trauma or neurological tumors, central nervous system diseases, psychiatric or endocrine disorders, or congenital anosmia. Additional exclusions included prior treatment with other monoclonal antibodies or biological agents within the previous 2 years, hypersensitivity to stapokibart or any of its excipients, active acute upper respiratory tract infection at screening, a diagnosis of chronic rhinosinusitis without nasal polyps, alcohol or drug abuse within the preceding 6 months, pregnancy or lactation, and severe hepatic or renal dysfunction. Participants received subcutaneous stapokibart (300 mg) every two weeks for 16 weeks.

**Table 1:** Characteristics of study patients with CRSwNP (*n* = 40).

Variable	CRSwNP (n=40)
Age, years (mean ± SD)	46.1 ± 15.4
Men, n (%)	24 (60.0%)
BMI (mean ± SD)	23.3 ± 3.28
Presence of AERD, n (%)	0%
Allergic rhinitis, n (%)	27 (67.5%)
Asthma, n (%)	18 (45.0%)
Duration of CRSwnp, years (mean ± SD)	6.64 ± 5.60
Endoscopic sinus surgery (ESS), n (%)	19 (47.5%)
SNOT-22 (mean ± SD)	38.2 ± 18.4
Total nasal VAS (mean ± SD)	6.51 ± 2.32
VAS for smell loss (mean ± SD)	8.60 ± 1.46
NPS (mean ± SD)	4.03 ± 1.70
Lund-Mackay CT score (mean ± SD)	16.5 ± 6.51
SSIT-16 (mean ± SD)	3.05 ± 2.98
Blood eosinophil count, eos/μL [median (minimum–maximum)]	380 (90, 4390)
Total IgE^*^, IU/mL[median (minimum–maximum)]	116 [27.7, 3000]

Data are presented as mean ± standard deviation (SD) for normally distributed variables, or median (range, minimum–maximum) for non-normally distributed variables. AERD, aspirin-exacerbated respiratory disease;SNOT-22 = 22-item Sino Nasal Outcome Test; VAS = Visual Analog Scale (0–10 cm); SSIT-16 = Sniffin’ Sticks-16 identification test. Subgroup stratification was based on the following predefined cut-off values: Total IgE: high (≥100 IU/mL) vs. low (<100 IU/mL); b Blood eosinophils: high (≥300 eos/μL) vs. low (<300 eos/μL); c Nasal Polyp Score (NPS): high (≥4) vs. low (<4). The distribution of patients across these subgroups was as follows: IgE (n=18 vs n=22), eosinophils (n=29 vs n=11), and NPS (n=27 vs n=13). *Data of total IgE were available in 26 patients.

### 2.2 Assessment of CRS severity

The severity of CRSwNP lesions was comprehensively determined through subjective assessment, objective examination, and laboratory testing. Subjective assessment included Total Nasal Symptom Visual Analog Scale (VAS, range: 0–10 cm) and 22-item Sino-Nasal Outcome Test (SNOT-22, range: 0–110 points), both evaluated at baseline, Week 2, 4, 8, and 16. Among them, the Total Nasal Symptom VAS score was used to assess the overall severity of nasal symptoms, and the SNOT-22 score was used to evaluate the impact of sinus symptoms on quality of life; higher scores indicated more severe symptoms and greater impairment of quality of life.

Objective assessment included Nasal Polyp Score (NPS, range: 0–8 points) and Lund-Mackay score (range: 0–24 points): NPS was measured by nasal endoscopy at baseline, Week 4, and 16 to assess the size and scope of nasal polyps; the Lund-Mackay score was used to evaluate the extent of sinus lesions based on high-resolution paranasal sinus CT, measured only at baseline and Week 16, with higher scores indicating more extensive lesions.

### 2.3 Assessment of olfactory function

Olfactory function assessment included subjective self-evaluation and objective testing. Subjective assessment was conducted at baseline, Week 2, 4, 8, and 16, while objective testing was performed at baseline, Week 4, and 16. Subjective self-evaluation used a VAS (range: 0–10 cm) to assess the degree of smell loss.

Objective testing was performed using the Sniffin’ Sticks-16 Identification Test (SSIT-16), which presented 16 odors at supra-threshold intensity in a multiple-choice format with a scoring range of 0–16 points. Olfactory function was classified according to the following criteria: normosmia (≥12 points), hyposmia (7–11 points), and anosmia (0–6 points) (26).

### 2.4 Statistical methods

Categorical variables were summarized as frequencies, while continuous variables were presented as means ± standard deviations. Intergroup comparisons were performed using the nonparametric Mann-Whitney U test, and *post-hoc* corrected P-values were used in multiple comparisons to control Type I error; repeated measurement data of olfactory function assessment were analyzed using the Friedman test to determine differences among different time points, and *post-hoc* P-value correction was also performed for intergroup multiple comparisons. After stratifying patients by baseline type 2 inflammatory markers (total IgE, eosinophils), NPS, previous sinus surgery history, allergic rhinitis (AR) status, and asthma status, Linear Mixed Models were used to evaluate the correlation between these stratifying factors and the improvement of olfactory function, with supplementary verification performed using the Wilcoxon rank-sum test. Subgroup stratification of type 2 inflammatory markers was performed according to predefined cut-off values. Peripheral blood eosinophils were dichotomized at 300 cells/μL based on the phase II subgroup analysis of stapokibart (25). Serum total IgE was stratified at 100 IU/mL following the type 2 inflammation criteria from the EPOS/EUFOREA 2023 guidelines (27). A two-tailed P<0.05 was considered statistically significant.

## 3 Result

### 3.1 Study population

A total of 54 patients meeting the inclusion criteria were initially enrolled, of whom 2 did not initiate monoclonal antibody therapy and 12 withdrew from treatment for personal reasons, resulting in a final sample of 40 patients with CRSwNP and moderate-to-severe olfactory dysfunction. All patients completed subjective symptom evaluations and olfactory function tests during the treatment period. The mean VAS score for olfactory loss was 8.60 ± 1.46, the mean score of SSIT-16 was 3.05 ± 2.98, and the mean NPS was 4.03 ± 1.70. Regarding the baseline demographic characteristics, 24 patients were male; the mean age of the enrolled patients was 46.1 ± 15.4 years. In terms of clinical history, 47.5% of the patients had a prior history of endoscopic sinus surgery (ESS), 67.5% were complicated with allergic rhinitis (AR), and 45.0% were complicated with asthma. Notably, none of the included patients had a history of aspirin-exacerbated respiratory disease (AERD). Detection results of type 2 inflammatory biomarkers indicated that the median blood eosinophil count was 380 eos/μL, and the median level of total immunoglobulin E (IgE) was 116 IU/mL, with a range of 27.7 to 3000 IU/mL (**Table 1**).

### 3.2 Results of olfactory improvement

**Figure 1:**
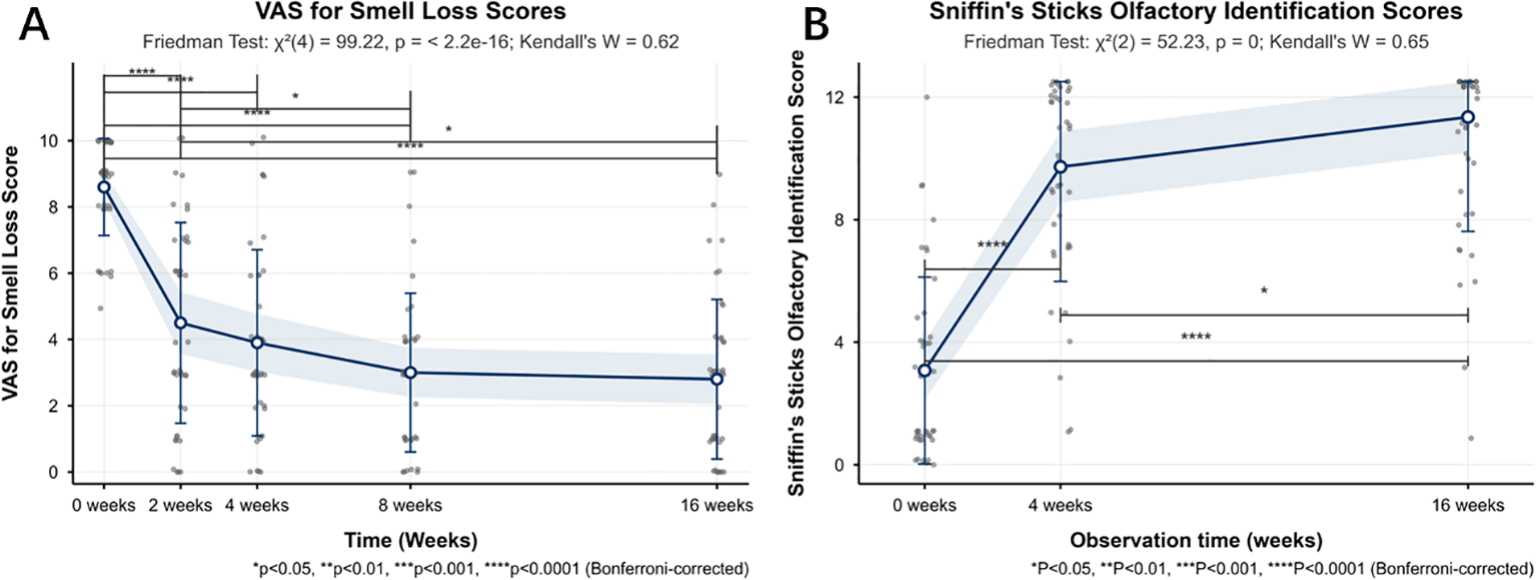
**(A)** Visual Analog Scale (VAS) scores for self-perceived smell loss, measured at 0, 2, 4, 8, and 16 weeks. Data are presented as group means (solid blue line) ± standard error of the mean (SEM; error bars), with individual participant data points overlaid (gray dots). Statistical analysis used the Friedman test: χ²(4) = 99.22, *p* < 2.2×10^-6^, Kendall’s *W* = 0.62 (moderate-to-strong effect size). Bonferroni-corrected pairwise significance levels are denoted as p < 0.05 (*), p < 0.01 (**), p < 0.001 (***), p < 0.0001 (****). **(B)** Sniffin’s Sticks Olfactory Identification Scores, evaluated at 0, 4, and 16 weeks. Data display group means (solid blue line) ± SEM (error bars) with overlaid individual participant data points (gray dots). Friedman test results: χ²(2) = 52.23, *p* < 2.2×10^-6^, Kendall’s *W* = 0.65. Bonferroni-corrected significance levels follow the same notation as **(A)**.

Improvement in olfactory function was already evident at Week 2 of treatment: VAS score for anosmia decreased from 8.60 ± 1.46 at baseline to 4.5 ± 3.03 (p<0.0001), and this ameliorative trend persisted with prolonged treatment, further declining to 2.8 ± 2.41 by Week 16 (p<0.0001) (**Figures 1A, B**). Consistently, objective olfactory test results aligned with the trajectory of subjective scores: the SSIT-16 score showed a gradual upward trend during treatment, reaching 9.72 ± 3.74 at Week 4 and further increasing to 11.35 ± 3.73 by Week 16 (p<0.0001). Friedman’s test for longitudinal comparisons validated these trends, revealing significant differences in VAS and SSIT-16 scores across time points (VAS: χ²(4)=99.22, p<2.2e-16, Kendall’s W = 0.62; SSIT-16: χ²(2)=52.23, p<0.0001, Kendall’s W = 0.65) (**Figures 1A, B**). These findings indicate that the therapeutic improvement in olfactory function is rapid, sustained, and statistically robust.

**Figure 2:**
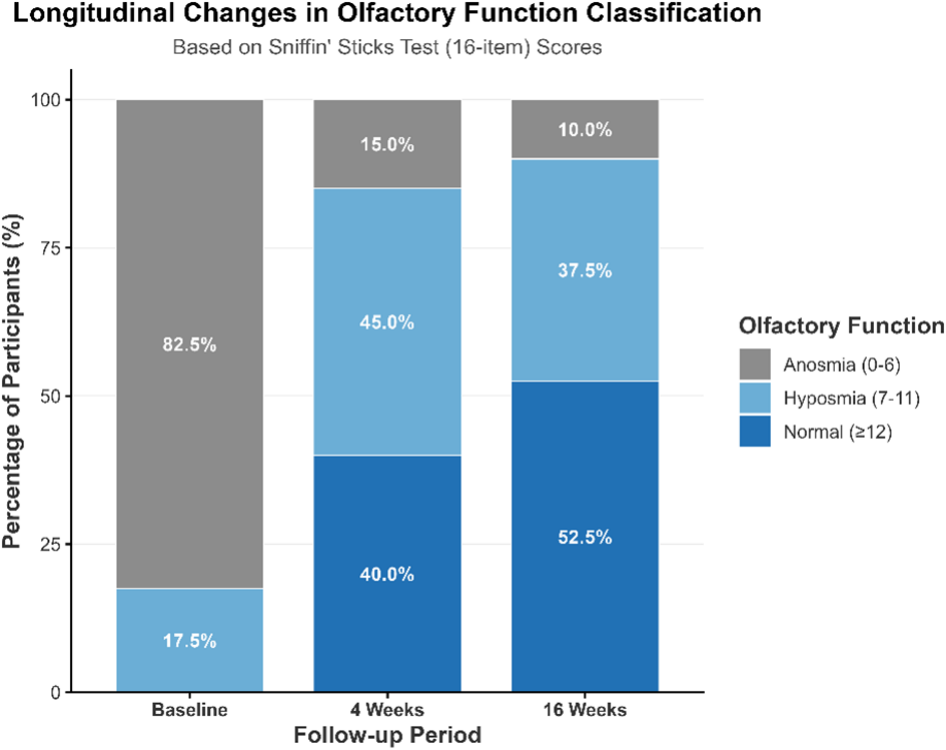
Longitudinal changes in olfactory function classification based on Sniffin’ Sticks-16 identification test. This stacked bar chart illustrates the percentage distribution of participants across three olfactory function categories at different follow-up time points (Baseline, 4 Weeks, 16 Weeks). Olfactory function was defined by Sniffin’s Sticks test scores: Anosmia (score range 0–6, gray segments), Hyposmia (score range 7–11, light blue segments), and Normal olfaction (score ≥12, dark blue segments).

To further quantify this improvement at the functional classification level, we analyzed olfactory function using the Sniffin’s Sticks-16 classification criteria (6), which categorizes olfactory function into three grades: normosmia (≥12 points), hyposmia (7–11 points), and anosmia (0–6 points). At baseline, 82.5% of patients presented with anosmia, and only 17.5% with hyposmia. Following 16 weeks of intervention, 52.5% of patients recovered to normosmia, 37.5% remained hyposmic, and the proportion of patients with anosmia significantly decreased to 10.0% (**Figure 2**).

### 3.3 Results of rhinitis symptom improvement in CRSwNP patients

#### 3.3.1 Subjective symptom improvements

**Figure 3:**
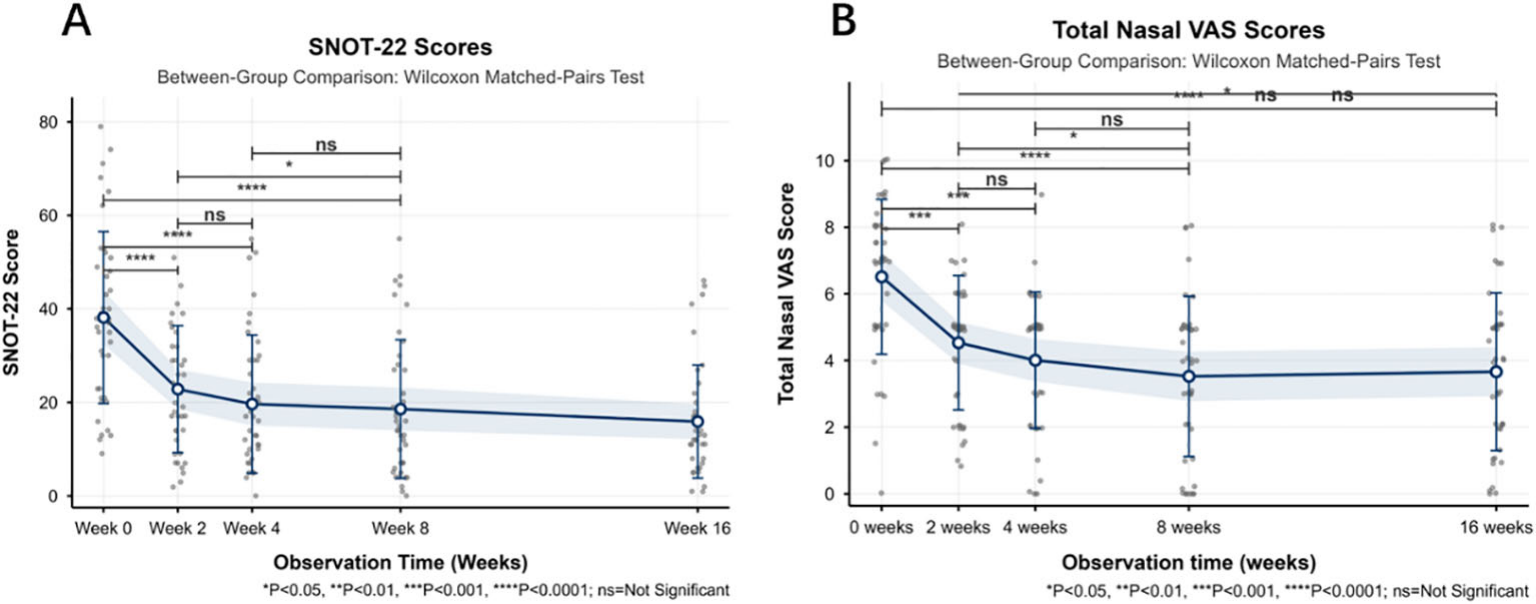
**(A)** Snot-22 score at 0, 2,4,8,16 weeks. Data: group mean (blue line) ± SEM (shaded area) with individual points; Paired statistical test significance: *p<0.05, **p<0.01, ***p<0.001, ****p<0.0001; ns=not significant. **(B)** Total nasal VAS score. Data: group mean (red line) ± SEM (shaded area) with individual points. Paired statistical test significance: *p<0.05, **p<0.01, ***p<0.001, ****p<0.0001; ns, not significant.

During treatment, subjective sino-nasal symptoms in patients were rapidly and sustainably alleviated. SNOT-22, a comprehensive measure of sino-nasal symptom severity, decreased from 38.15 ± 18.39 at baseline to 22.82 ± 13.58 by Week 2, with further reductions to 19.65 ± 14.77 (Week 4), 18.58 ± 14.81 (Week 8), and 15.9 ± 12.08 (Week 16) (all p<0.0001 vs. baseline; **Figure 3A**).

A concurrent trend toward improvement was observed for the total nasal symptom VAS score: it declined from 6.51 ± 2.32 at baseline to 4.53 ± 2.02 by Week 2 (p=4×10^-4^), further decreased to 3.52 ± 2.4 by Week 8, and remained at 3.66 ± 2.37 by Week 16 (all post-treatment time points p<0.0001 vs. baseline; **Figure 3B**).

#### 3.3.2 Objective pathological improvements

**Figure 4:**
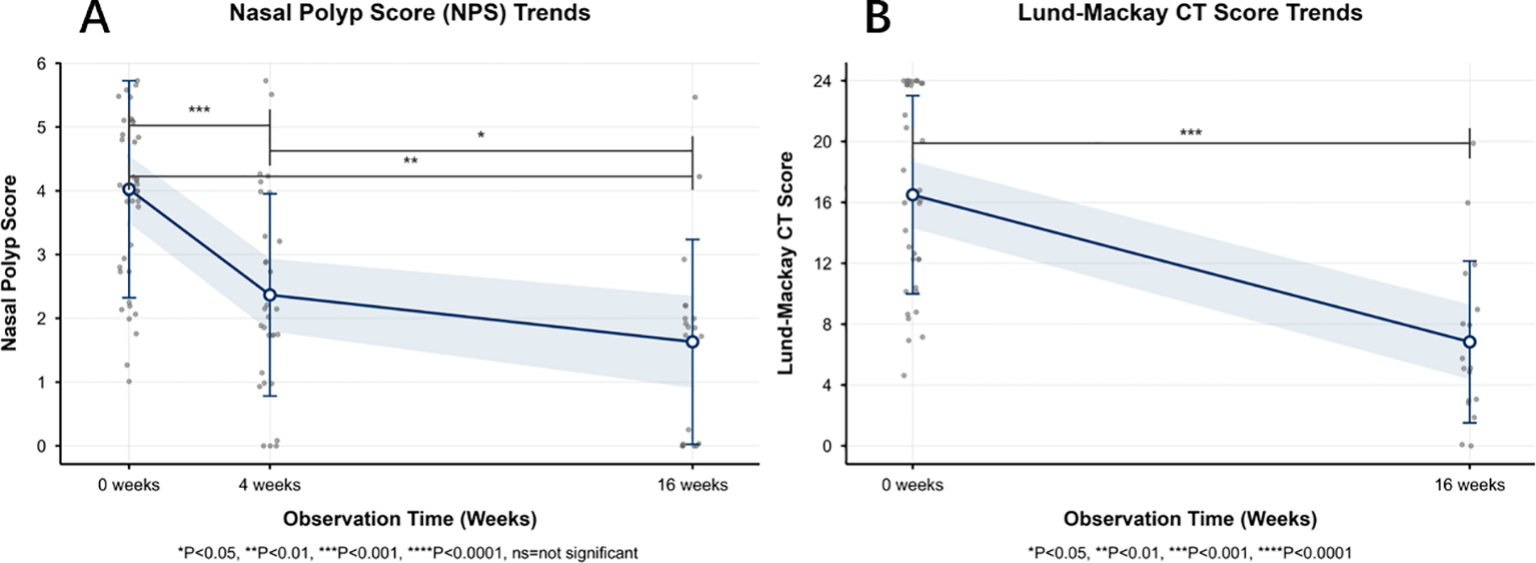
**(A)** Nasal Polyp Score (NPS) at 0, 4, 16 weeks. Presented as mean (blue line) ± SEM (shaded area) with individual points. Paired statistical test significance: *p<0.05, **p<0.01, ***p<0.001, ****p<0.0001; ns, not significant. **(B)** Lund-Mackay CT scores over follow-up. Presented as mean (blue line) ± SEM (shaded area) with individual points. Paired test significance as in **(A)**.

Objective assessments confirmed significant regression of nasal and sinus pathology in CRSwNP patients. The NPS, a metric for quantifying polyp burden, decreased from 4.03 ± 1.70 at baseline to 2.37 ± 1.59 by Week 4 (p<0.0001), and further declined to 1.63 ± 1.61 by Week 16 (p<0.05 vs. baseline; **Figure 4A**), indicating progressive reduction in polyp size and severity. Consistently, the Lund-Mackay CT score (a measure of sinus inflammation and mucosal thickening) decreased from 16.50 ± 6.51 at baseline to 6.83 ± 5.32 by Week 16 (p<0.001; **Figure 4B**), demonstrating marked resolution of sinus inflammatory lesions.

#### 3.3.3 Systemic inflammatory marker changes

**Figure 5:**
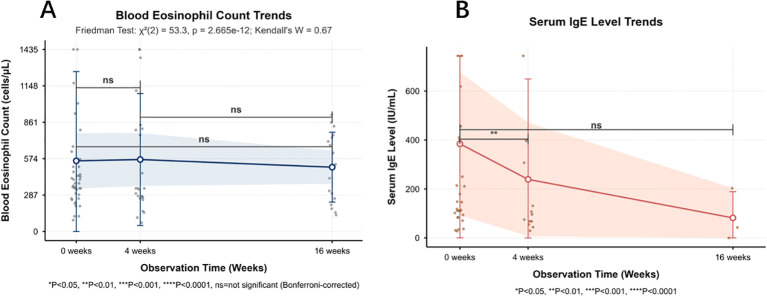
**(A)** Blood eosinophil count at 0, 4, 16 weeks. Data: group mean (blue line) ± SEM (shaded area) with individual points; ns, not significant. **(B)** Serum IgE level over follow-up. Data: group mean (red line) ± SEM (shaded area) with individual points. Paired statistical test significance: *p<0.05, **p<0.01, ***p<0.001, ****p<0.0001; ns, not significant.

Pairwise comparisons of peripheral blood eosinophil counts between each time point and baseline were statistically non-significant (**Figure 5A**), suggesting no clinically meaningful changes in this parameter. However, serum IgE levels decreased significantly: from 384.33 ± 759.14 at baseline to 239.12 ± 410.45 by Week 4 (p <0.01), and further to 81.92 ± 106.99 by Week 16 (**Figure 5B**).

### 3.4 Post-treatment olfactory improvement is not influenced by baseline stratification factors

**Figure 6:**
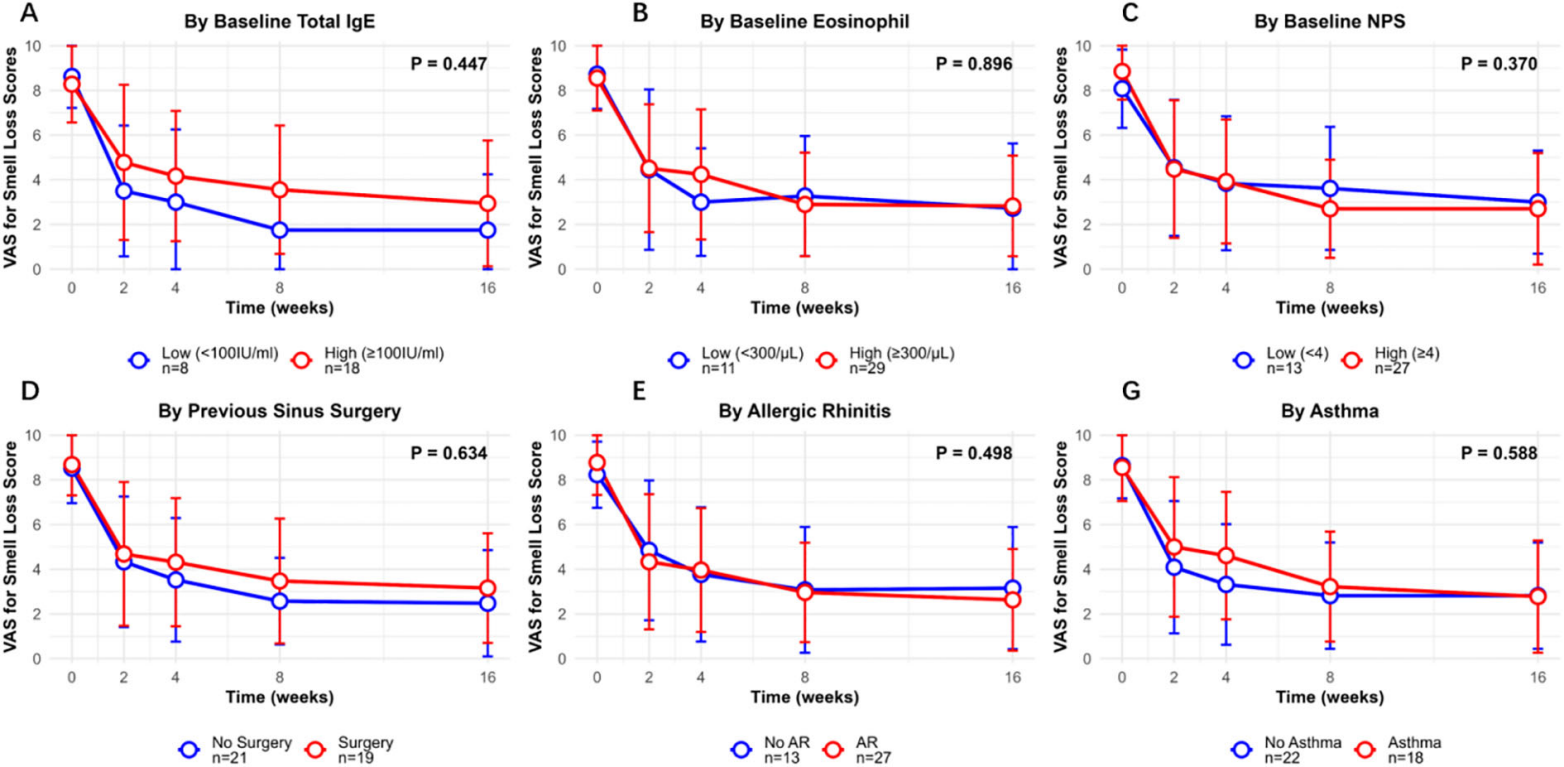
**(A–G)** Subgroup analysis of VAS score for smell loss changes using linear mixed-effects models, with p-values representing time-by-group interaction effects. Results show consistent treatment response trends across all subgroups, with no significant heterogeneity detected (all p > 0.05). Error bars represent standard error of the mean.

To evaluate the consistency of stapokibart efficacy in CRSwNP patients with different baseline characteristics, we conducted subgroup analyses stratified by key clinical features, including total IgE level, peripheral blood eosinophil count, baseline NPS, history of prior sinus surgery, comorbid AR, and comorbid asthma. Two outcomes were assessed: subjective VAS for smell loss (**Figures 6A–G**) and objective SSIT-16 (**Table 2**). For VAS, linear mixed-effects models were used, focusing on evaluating time-by-subgroup interaction effects. Results showed no significant interaction effects across all subgroups (p > 0.05), indicating highly consistent trends in VAS score improvement among subgroups with different baseline characteristics and no heterogeneous treatment responses observed.

**Table 2:** Comparison of Sniffin’ Sticks-16 identification test scores among subgroups stratified by clinical characteristics.

				Baseline		Week 4		Week 16	
Group	Criteria	Subgroup	n	Baseline	P (Baseline)	Δ4w	P (Δ4w)	Δ16w	P (Δ16w)
Total IgE	*Serum total IgE ≥100 IU/mL vs <100 IU/mL*	High IgE	18	3.50 ± 3.45	0.628	5.78 ± 4.65	0.118	6.94 ± 4.93	0.070
Total IgE	*Serum total IgE ≥100 IU/mL vs <100 IU/mL*	Low IgE	8	2.75 ± 2.82		9.12 ± 4.12		10.75 ± 3.58	
Eosinophil	*Peripheral eosinophil count ≥0.3×10^9^/L vs <0.3×10^9^/L*	High Eos	29	2.97 ± 2.61	0.767	6.48 ± 4.73	0.808	8.45 ± 4.49	0.412
Eosinophil	*Peripheral eosinophil count ≥0.3×10^9^/L vs <0.3×10^9^/L*	Low Eos	11	3.73 ± 3.98		6.91 ± 4.23		7.09 ± 4.61	
Nasal Polyposis Score	*NPS ≥4 vs <4 at baseline*	High NPS	27	2.85 ± 2.76	0.340	7.81 ± 4.10	0.024	8.26 ± 4.43	0.761
Nasal Polyposis Score	*NPS ≥4 vs <4 at baseline*	Low NPS	13	3.85 ± 3.51		4.08 ± 4.52		7.69 ± 4.84	
Prior Sinus Surgery	*History of sinus surgery vs no history*	No Surgery	19	3.42 ± 3.24	0.665	6.16 ± 4.51	0.625	9.05 ± 4.25	0.166
Prior Sinus Surgery	*History of sinus surgery vs no history*	Surgery	21	2.95 ± 2.85		7.00 ± 4.65		7.00 ± 4.65	
Allergic Rhinitis	*With allergic rhinitis vs without*	AR	27	3.33 ± 2.80	0.311	6.56 ± 4.48	0.862	8.15 ± 4.47	0.885
Allergic Rhinitis	*With allergic rhinitis vs without*	No AR	13	2.85 ± 3.51		6.69 ± 4.87		7.92 ± 4.77	
Asthma	*With asthma vs without asthma*	Asthma	18	2.18 ± 1.85	0.171	7.24 ± 3.80	0.418	9.00 ± 4.08	0.255
Asthma	*With asthma vs without asthma*	No Asthma	22	3.91 ± 3.50		6.13 ± 5.06		7.39 ± 4.77	

Data for the Sniffin’ Sticks-16 Identification Test (SSIT-16) are presented as mean ± standard deviation (SD). Baseline = mean value of baseline scores from the SSIT-16, which assesses participants’ initial olfactory identification ability before intervention; Δ4w = mean change in SSIT-16 scores from baseline to Week 4 post-intervention; Δ16w = mean change in SSIT-16 scores from baseline to Week 16 post-intervention (a positive value indicates an increase in SSIT-16 scores, i.e., improved olfactory identification ability, compared with baseline). Intergroup comparisons for these parameters were performed using Wilcoxon rank-sum test, with a P value < 0.05 considered statistically significant.

At baseline, between-subgroup comparisons of SSIT-16 scores revealed no statistical differences, suggesting well-balanced baseline olfactory function across all subgroups. Wilcoxon tests were used to analyze changes from baseline and between-subgroup differences in SSIT-16 scores. Results demonstrated that all subgroups showed significant improvements in SSIT-16 scores at Week 4 and Week 16 compared with baseline. For between-subgroup differences in the magnitude of improvement: only the high baseline NPS subgroup exhibited a statistically significant difference in improvement compared with the low NPS subgroup at Week 4 (p = 0.024), but this difference resolved by Week 16 (p = 0.761). No significant between-subgroup differences were noted at Week 4 (p > 0.05) or Week 16 (p > 0.05) for the remaining subgroups (**Table 2**).

In conclusion, stapokibart consistently improves both subjective olfactory experience and objective olfactory function in CRSwNP patients, regardless of baseline type 2 inflammatory status (reflected by total IgE and eosinophil levels), nasal polyp burden, history of prior sinus surgery, or comorbidities. The absence of subgroup-specific differences in therapeutic benefits supports its broad applicability in CRSwNP patients with olfactory dysfunction.

## 4 Discussion

This study provides robust real-world evidence for the efficacy of stapokibart in treating moderate-to-severe OD associated with CRSwNP. Our core findings demonstrate that stapokibart induces a rapid and sustained recovery of olfactory function: self-reported VAS scores for smell loss significantly improved as early as Week 2, and by Week 16, 52.5% of the total cohort had achieved normosmia.

In addition, an intriguing finding was observed in the subgroup analysis of the present study: the olfactory improvement induced by stapokibart, as reflected by both subjective olfactory scores and objective olfactory functional assessments, was independent of baseline peripheral type 2 inflammatory biomarker levels (**Figure 6**, **Table 2**). As a humanized monoclonal antibody targeting IL-4Rα, stapokibart specifically blocks the IL-4/IL-13 signaling axis, a pivotal pathway driving type 2 inflammation. Accordingly, we initially hypothesized that patients with elevated baseline type 2 inflammation would derive greater therapeutic benefits; however, the present findings failed to validate this hypothesis.

We postulate that CRSwNP-associated olfactory dysfunction arises from the interplay of multiple pathogenic factors, and systemic type 2 inflammatory burden cannot accurately predict the severity or recovery trajectory of olfactory impairment. Mucosal edema, mucus retention, and nasal polyp occupation lead to conductive olfactory dysfunction by obstructing the access of odorant molecules to the olfactory cleft. By suppressing type 2 inflammation, stapokibart rapidly alleviates mucosal edema and reduces polyp size, thereby relieving mechanical obstruction. Nevertheless, mechanical obstruction is not the sole dominant mechanism underlying olfactory dysfunction. Surgical polypectomy alone, despite restoring ventilation of the olfactory cleft, often fails to achieve complete olfactory recovery (26, 28). Notably, marked olfactory impairment can also occur in patients with chronic rhinosinusitis without nasal polyps.

Emerging preclinical evidence indicates that olfactory damage is further attributed to local inflammation-mediated alterations in neural function. Preclinical murine studies have demonstrated that intranasal administration of IL-4 (but not IL-13) directly suppresses calcium responses of OSNs to odorants and impairs olfactory signal transduction. Meanwhile, IL-4 activates macrophages, mast cells and NK cells, disrupts neuroimmune homeostasis, and triggers neuroinflammation, ultimately resulting in olfactory decline independent of structural destruction of the olfactory epithelium (14, 23). This mechanistic insight provides a plausible interpretation for our subgroup results. In the objective olfactory subgroup analysis (**Table 2**), patients in the high NPS subgroup exhibited more prominent olfactory improvements at Week 4, which was highly consistent with rapid polyp regression and prompt resolution of mechanical obstruction. By contrast, the low NPS subgroup showed a relatively mild early therapeutic response; nevertheless, the magnitude of olfactory recovery eventually converged between the two groups with prolonged treatment, with no statistically significant intergroup difference.

These findings suggest that baseline polyp burden may influence the onset rate of olfactory recovery rather than the ceiling of ultimate therapeutic efficacy. From a clinical perspective, this implies that treatment should not be prematurely discontinued for patients with low systemic type 2 inflammation, mild polyp burden and slow early response. Sustained inhibition of the IL-4 pathway may enable patients across different clinical phenotypes to attain comparable olfactory improvement outcomes.

Dupilumab, another approved agent targeting IL-4Rα, has been well documented to exert robust beneficial effects on olfactory function in patients with CRSwNP (29, 30). Transient peripheral blood eosinophilia is a frequently reported clinical event following dupilumab administration (31). Conversely, no such eosinophilic fluctuation was observed during stapokibart treatment in our cohort, with overall eosinophil levels remaining stable without obvious transient elevation. This discrepancy may be partially attributed to the relatively small sample size of the present study, which cannot rule out the possibility of unobserved mild and transient eosinophil fluctuations. Furthermore, dupilumab and stapokibart bind to non-overlapping epitopes on IL-4Rα: dupilumab targets the distal region of the receptor, whereas stapokibart binds closer to the ligand-binding domain, which may also contribute to the differential hematological profiles between the two agents (24).

Despite the promising results, this study has several limitations. First, the definition of type 2 inflammation was based solely on peripheral blood biomarkers rather than the comprehensive EPOS/EUFOREA 2023 criteria, which include tissue eosinophilia (≥10 eosinophils/hpf), blood eosinophilia (≥150 eosinophils/mm³), or total IgE ≥100 IU/mL (28). Considering the potential mucosal damage and low patient tolerance of nasal tissue biopsy, local tissue eosinophils were not evaluated in the present study. However, tissue eosinophilia has been strongly associated with disease severity and treatment outcomes (29), indicating that further stratification based on local inflammatory phenotypes is warranted in future investigations. Second, the type and extent of previous endoscopic sinus surgery varied widely among patients and were not standardized, which may have an important impact on baseline olfactory function and treatment response (30). Third, this was a single-arm study without a control group, making it difficult to eliminate confounding factors such as the placebo effect and natural disease course. Finally, the relatively small sample size (n = 40) may limit the statistical power of subgroup analyses and the generalizability of the conclusions. Finally, the 16-week observation period is insufficient to evaluate the long-term durability of olfactory improvement or potential late-onset adverse effects associated with stapokibart. Future research should address these gaps through large-scale, randomized controlled trials (RCTs) with extended follow-up periods to confirm the sustained efficacy and safety of stapokibart.

## 5 Conclusion

In conclusion, this study demonstrates that stapokibart, an IL-4Rα inhibitor, provides rapid and sustained olfactory improvement in CRSwNP patients, with consistent efficacy across subgroups with varying baseline characteristics. The observed benefits likely stem from the suppression of neuroinflammation and protection of olfactory neurons via IL-4/IL-13 pathway blockade.

## Data Availability

The raw data supporting the conclusions of this article will be made available by the authors, without undue reservation.
